# Wnt Inhibitors and Bone Mineral Density in Patients with Graves’ Disease Treated with Antithyroid Drugs: A Preliminary Prospective Study

**DOI:** 10.3390/metabo12080711

**Published:** 2022-07-29

**Authors:** Dunja Mudri, Tomislav Kizivat, Ivica Mihaljević, Ines Bilić Ćurčić

**Affiliations:** 1 Clinical Institute for Nuclear Medicine and Radiation Protection, University Hospital Osijek, 31000 Osijek, Croatia; dmudri@mefos.hr (D.M.); tkizivat@mefos.hr (T.K.); ivanm2712@gmail.com (I.M.); 2 Department for Nuclear Medicine and Oncology, Faculty of Medicine Osijek, J.J. Strossmayer, University of Osijek, 31000 Osijek, Croatia; 3 Academy of Medical Sciences of Croatia, 10000 Zagreb, Croatia; 4 Department of Endocrinology, University Hospital Osijek, 31000 Osijek, Croatia; 5 Department of Pharmacology, Faculty of Medicine Osijek, J.J. Strossmayer, University of Osijek, 31000 Osijek, Croatia

**Keywords:** hyperthyroidism, sclerostin, Dickkopf-1 protein, bone density, biomarkers

## Abstract

This study aimed to investigate the association of Wnt inhibitors with thyroid hormones, bone turnover markers, and bone mineral density (BMD) in patients with newly diagnosed Graves’ disease (GD) at the beginning of the antithyroid treatment and after a follow-up period of one year. The study included 37 patients with newly diagnosed GD who were treated with antithyroid drugs (ATD). At baseline and after one year, thyroid hormones and thyroid-stimulating hormone (TSH), serum concentrations of sclerostin, and Dickkopf-1 (DKK1) were measured by an enzyme-linked immunosorbent assay (ELISA). In addition, BMD was measured by dual-energy X-ray absorptiometry (DXA), and markers of bone turnover including osteocalcin (OC), beta-cross laps (β-CTX), and deoxypyridinoline (DPD) were determined. After one year of ATD therapy sclerostin levels were significantly decreased (*p* < 0.001), whereas DKK1 levels were significantly increased (*p* = 0.01). In addition, BMD of the lumbar spine, total hip, and femoral neck was significantly improved (*p* < 0.001), accompanied by an increase in OC, β-CTX, and DPD concentrations (*p* < 0.001). At baseline, sclerostin levels were positively associated with free triiodothyronine (FT3). Following ATD therapy, a positive correlation was observed between FT3 and DKK1 (*p* = 0.003), whereas a negative correlation was found between TSH and DKK1 (*p* = 0.04). Correlation analysis demonstrated no association of the sclerostin and DKK1 with other bone remodeling biomarkers OC, β-CTX, or DPD. Also, no significant correlation between sclerostin or DKK1 and T-score or BMD of the lumbar spine, hip, and femoral neck was observed at both time points. Conclusion: Observed differences in sclerostin and DKK1 serum following GD treatment indicate involvement of Wnt inhibitors in the etiopathogenesis of bone loss associated with hyperthyroidism. Furthermore, both sclerostin and DKK1 are involved in the reversal of changes in bone metabolism following ATD therapy, thus presenting potentially valuable bone remodeling markers worth further investigation.

## 1. Introduction

Graves’ disease (GD) is the most common cause of hyperthyroidism. It is an autoimmune disease in which circulating antibodies bind to the thyroid-stimulating hormone (TSH) receptor (TSH-R) on the surface of thyroid follicular cells [1]. The thyroid is stimulated and excessively produces thyroid hormones, both thyroxine (T4) and triiodothyronine (T3), which are released in circulation. Most of the excreted hormones are bound to plasma proteins and the rest circulate unbound, freely, so they are called free T4 (FT4) and free T3 (FT3) [2]. Usually, there are 20–50 cases per 100,000 people per year [3]. Women aged between 30 and 60 are predominantly affected [4]. GD can be treated by antithyroid drugs (ATD), radioiodine therapy (RITh), and thyroid surgical operation [5].

One of the consequences of GD can be accelerated bone metabolism and shortened bone remodeling cycle by 50% [6]. A balance between bone formation and bone resorption is disrupted in a way that bone resorption exceeds bone formation leading to bone density decrease, i.e., osteopenia or osteoporosis. Loss of bone density in adults yields 10–20%, mostly involving cortical bone [1]. Osteopenia is an indicator of fracture risk. Osteoporosis is a disease characterized by loss of bone mass and deterioration of bone microarchitecture, leading to fracture and disability. The goal of ATD treatment is to achieve a euthyroid state. Consequently, it can be expected for bone density to improve.

In the last couple of decades, diseases characterized by bone density disorders have been extensively investigated [7]. One of the key factors in maintaining bone homeostasis is the wingless/integrated (Wnt) signaling pathway. It consists of two major types due to its complexity: (1) the Wnt/beta-catenin pathway, which is also called the canonical Wnt pathway, and (2) the noncanonical pathway, which includes Wnt/planar cell polarity (PCP) and Wnt calcium (Ca^2+^) pathways, respectively [8,9]. The canonical Wnt pathway is important for all bone cells involved in bone remodeling, i.e., osteoblasts, osteoclasts, and osteocytes [10]. Wnt stimulation leads to bone density improvement and, in contrast, its inhibition leads to bone density reduction [9]. Therefore, Wnt inhibitors have become a focus of research, especially sclerostin and Dickkopf-1 (DKK1).

Sclerostin is a glycoprotein encoded by the SOST gene [11]. It is secreted mostly in osteocytes [12,13], but it has also been found in osteoblasts, osteoclasts, chondrocytes, odontoblasts, and cementocytes [14]. Moreover, SOST could be transcribed in bone marrow, cartilage, kidney, liver, lungs, and pancreas [15]. Osteocytes are embedded within the mineral matrix in bone [16]. Sclerostin influences bone formation via osteoblast activity inhibition and promotion of their apoptosis leading to bone formation inhibition [17].

DKK1 is a glycoprotein and a part of a family consisting of four members (Dickkopf-1–4) [18]. It is secreted by osteocytes, but it is also expressed in skin, endothelium, prostate, placenta, and platelets (and to a lesser degree in some other tissues [19,20]. Within bone, it inhibits osteoblasts’ development and activity. Hence, DKK1’s increased concentration can impair osteoblast activity and cause bone loss [21]. Recently, a new drug for the treatment of severe osteoporosis was approved, a sclerostin inhibitor romosozumab [22].

So far, only a few studies have investigated the interrelationship between hyperthyroidism, the Wnt pathway, and bone remodeling. To our knowledge, no studies have investigated the associations between elevated concentrations of thyroid hormones and DKK1 in humans, and only a few have investigated associations between elevated concentrations of thyroid hormones and sclerostin [23,24,25,26,27,28].

Since hyperthyroidism influences bone density, our study aimed to investigate the association of circulating levels of Wnt inhibitors with thyroid hormone levels, bone mineral density (BMD), and other markers of bone turnover in newly diagnosed GD patients at the initiation of the ATD treatment and after a follow-up of one year.

## 2. Results

Thirty-seven patients completed the study. Four were males (11%) and thirty-three were females (89%), aged between 22 and 77 years (with an average age of 47). Median body mass index (BMI) was 24.4 kg/m^2^. Baseline characteristics are summarized in Table 1.

The median age of menopause onset was 50 years. All the 33 women had a regular menstrual cycle, and, among them, 16 reached menopause. None of the women had a hysterectomy and only one had an adnexectomy at the age of 33. During the follow-up period, 20 patients were treated with vitamin D supplements due to the vitamin D deficiency diagnosed at baseline.

Following GD treatment, a significant decrease in thyroid hormones concentrations and anti-thyroid stimulating hormone receptor antibodies (anti-TSH-R) was achieved (*p* < 0.001 for all). Also, a significant increase in TSH concentrations was noted (*p* < 0.001), as shown in Table 2.

Serum sclerostin levels were significantly higher in the hyperthyroid state compared to control measurements (*p* < 0.001). In contrast, there was a rise in serum DKK1 levels at control compared to the baseline point (*p* = 0.01), Table 3.

After treatment of GD, a significant decrease in osteocalcin (OC), beta-cross laps (β-CTX), and deoxypyridinoline (DPD) levels was observed (*p* < 0.001), as shown in Table 3.

T-scores and BMD measurements of the lumbar spine, total hip, and femoral neck were significantly higher at euthyroid compared to the hyperthyroid state (*p* < 0.001), as shown in Table 4.

Before therapy initiation, 16 participants (43%) had a normal dual-energy X-ray absorptiometry (DXA) score of the lumbar spine, osteopenia was present in 20 participants (54%), and only 1 participant had osteoporosis, as shown in Table 5. At the control visit, 25 (67%) participants had normal T-scores, 11 (30%) presented with osteopenia, and only 1 had osteoporosis marking a significant improvement of lumbar bone density (*p* = 0.007) achieved with ATD therapy, Table 5.

Regarding hip bone density, 24 participants (65%) had normal findings, osteopenia was noted in 12 (32%), and osteoporosis in only 1 subject. After one year, normal hip bone density was present in 27 (73%) participants, osteopenia in 10 (27%), while none had osteoporosis, a difference not reaching statistical significance, as shown in Table 5.

At the baseline, a positive correlation of FT4 and FT3 with OC (*p* = 0.02, 0.04 respectively), β-CTX (*p* = 0.03, 0.02 respectively), and DPD (*p* < 0.001 for both) was found, as well as between anti-TSH-R and DPD (*p* = 0.01). Furthermore, the T-score of the lumbar spine, hip, and femoral neck negatively correlated with OC (*p* = 0.006, 0.002, 0.01 respectively) and β-CTX (*p* = 0.02, 0.02, 0.03 respectively). BMD of the lumbar spine, hip, and femoral neck also negatively correlated with OC (*p* = 0.01, 0.007 and 0.02 respectively) and β-CTX (*p* = 0.02, 0.02 and 0.04 respectively). Sclerostin levels were positively associated with FT3 (*p* = 0.19), as shown in Table 6.

At the control visit, a positive correlation was observed between FT4 and DPD (*p* = 0.003) and FT3 and DKK1 (*p* = 0.003), whereas a negative correlation was found between TSH and DKK1 (*p* = 0.04). In addition, a negative correlation existed between the T-score of the lumbar spine, hip, and femoral neck and OC (*p* = 0.03, 0.02 and 0.02 respectively), as well as between the BMD of the lumbar spine, hip, femoral neck, and OC (*p* = 0.08, 0.004 and 0.001 respectively). β-CTX negatively correlated with the T-score of the lumbar spine (*p* = 0.006), as shown in Table 7.

No significant correlation was observed between sclerostin or DKK1 and the T-score or BMD of the lumbar spine, hip, and femoral neck at both time points, as shown in Table 6 and Table 7.

## 3. Discussion

The negative impact of hyperthyroidism on bone metabolism and bone density has been well established [29,30]. The Wnt signaling pathway and its inhibitors sclerostin and DKK1 play an important role in the maintenance of bone health [21,31,32]. However, the association of Wnt inhibitors with BMD and bone turnover markers in hyperthyroidism has not yet been established.

This is the first longitudinal prospective study investigating the role of sclerostin and DKK1 in patients newly diagnosed with GD and treated with ATD. We found that reaching euthyroid status in GD patients results in a significant decrease in sclerostin and an increase in DKK1 concentrations. Moreover, an improvement of bone density in the lumbar spine, total hip, and femoral neck, as well as markers of bone remodeling, was observed.

Data regarding the association between hyperthyroidism and sclerostin are scarce, and there are only a few studies carried out in humans. In two studies published by the same group, sclerostin levels were determined in patients of both genders diagnosed with GD or multinodular goiter at the time of diagnosis and 6–10 weeks after ATD initiation. Our results are in line with those studies, since a significant decrease in sclerostin concentrations was measured along with achieving a euthyroid state [25,26].

The results of investigations assessing sclerostin in the serum of hyperthyroid patients and euthyroid controls vary. In a study published by Saritekin et al. [27], sclerostin levels were determined before ATD initiation in two groups of patients, hyperthyroid and control (24 subjects in each group). Sclerostin serum concentrations did not differ significantly between the two groups. Mihaljević et al. [28] also compared sclerostin levels in patients with GD and toxic nodular goiter, as well as controls; however, both groups had fewer participants, 10 in each group. They found considerably greater serum sclerostin concentrations in the hyperthyroid group compared to controls, which contradicts previous findings.

To date, serum DKK1 concentrations in hyperthyroid human subjects have not been investigated. Tsourdi et al. [23] investigated DKK1 and sclerostin levels in mice. Serum DKK1 concentrations were lower and serum sclerostin concentrations were higher in hyperthyroid animals, which is in line with our findings. In a study published by the same group of researchers [24], the role of DKK1 in hyperthyroidism-induced bone abnormalities in mice with global or osteocyte-specific DKK1 deletion was investigated. In neither of these groups, loss of DKK1 function was not sufficient to fully reverse bone changes caused by hyperthyroidism.

Our results regarding the behavior of sclerostin and DKK1 serum concentrations in hyperthyroidism are consistent with earlier findings. We could hypothesize that thyroid hormones regulate these two Wnt inhibitors differently and that they may have diverse effects on hyperthyroidism-induced bone alterations. Another explanation is that they complement each other [33], with the canonical Wnt pathway increasing sclerostin and decreasing DKK1 expression in hyperthyroidism to avoid excessive osteoblast differentiation. Inverse DKK1 concentrations in serum could represent decreased bone formation [21], but also a counterbalance to higher sclerostin levels aiming to limit the impact of sclerostin on bone tissue. Opposite concentrations of sclerostin and DKK1 are known from previous investigations [34]. 

An additional important factor to be considered is the influence of ATD on bone tissue changes. The bone remodeling process is prolonged, and bone formation and bone resorption become more balanced, so concentrations of bone remodeling markers and bone density improve. In accordance, levels of sclerostin and DKK1 in serum also change. Increased concentration of DKK1 probably represents higher bone formation, while decreased sclerostin concentration is probably due to its lower synthesis in osteocytes and decreased biochemical stimuli.

Bone turnover was assessed by analyzing bone formation marker OC and bone resorption markers β-CTX and urine DPD. All analyzed biomarkers OC, β-CTX, and DPD were significantly higher in the hyperthyroid state as opposed to the control measurement in the euthyroid state. The same result was reported in a previously published study [35], which included only male patients and controls, while in our study mainly female patients were investigated. In other studies, bone remodeling markers were investigated in the hyperthyroid state compared to controls [28,36]. In the above-mentioned study by Mihaljević et al. [28], significantly higher OC and β-CTX levels in hyperthyroid patients than in controls were noted.

Patients with GD and multinodular toxic goiter were also included in the study conducted by Akalin et al. [34], with a total of 26 patients and 20 controls. Hyperthyroid individuals had significantly higher serum OC and urine DPD values than controls. Our findings regarding OC are in accordance with the results of previous studies [37], including individuals with multinodular goiter and toxic adenoma, as well as GD patients. Another study revealed a substantial increase in OC levels during hyperthyroidism treatment, mainly in premenopausal women. This study included 13 patients with newly diagnosed GD, 7 of whom were postmenopausal women and 4 premenopausal women, as well as 2 men [38]. OC levels steadily declined to reach pretreatment levels later in the study.

In the current investigation, control BMD and T-score values of the lumbar spine, total hip, and femoral neck were considerably improved when compared to the hyperthyroid state confirming previously published results. However, subjects included varied greatly between studies, some including only men and premenopausal women, some postmenopausal women, whereas pre- and postmenopausal women and men were included in our study. In addition, some studies were conducted on patients with toxic multinodular goiter along with GD, treated with ATD, total thyroidectomy, and RITh [30,39,40,41,42,43].

In a study of 25 patients with thyrotoxicosis who were treated with ATD, subtotal thyroidectomy, or RITh, Krolner et al. [39] found an increase in lumbar bone mineral content (BMC) following antithyroid medication. Wakasugi et al. [40] examined BMD of the lumbar spine, femoral neck, and Ward’s triangle in 15 men and women with newly diagnosed GD and treated with ATD for 4 to 20 months. The control BMD measurements showed significant improvement compared to pretreatment findings. In addition, Belsing et al. [41] found significant improvement in BMD in premenopausal women with GD after 18 ± 3 months of ATD treatment. In a study investigating 30 men and premenopausal women with GD, an improvement in BMD of the lumbar spine and femur was observed after ATD treatment. Another study including postmenopausal women (*n* = 42) monitored for 24 months revealed that 26% of osteoporotic patients had recovered from their condition. Furthermore, Nicolaisen et al. [30] followed up on 46 women aged 20 to 85 years who had been diagnosed with hyperthyroidism as a result of GD or toxic nodular goiter and were treated with ATD, complete thyroidectomy, or RITh. Hip and lumbar spine assessed by DXA showed an increase one year after achieving a euthyroid state.

In our study, at the time of diagnosis of GD, only one of our patients had osteoporosis of the lumbar spine, which also persisted at control measurement. Other patients exhibited osteopenia or normal lumbar spine and hip bone density. At baseline, osteopenia of the lumbar spine was more frequent than osteopenia of the hip. The duration of the hyperthyroid state could be a possible explanation for these findings. Due to the availability of thyroid hormone laboratory tests and widely available healthcare, it is feasible that subjects were diagnosed with GD in a relatively short period before the hyperthyroid state could have a significant impact on BMD.

Thyroid hormones, TSH, anti-TSH-R, and DXA measurements of the lumbar spine, total hip, and femoral neck were correlated with bone turnover markers and Wnt inhibitors at baseline and one year after ATD treatment. Before initiating ATD, a significant positive correlation between all bone turnover markers, OC, DPD, and β-CTX and both FT4 and FT3 was found, which is in agreement with the results of the previously mentioned study [35].

Various markers of bone remodeling and thyroid function were investigated in other studies. Akalin et al. [36] demonstrated a positive association between urine DPD levels and serum FT4 and FT3; however, the correlation between OC and thyroid hormone concentrations was absent as opposed to our findings [36]. In our study, only DPD positively correlated with anti-TSH-R, as opposed to another study reporting a negative correlation between anti-TSH-R and OC [44]. T-score and BMD of the lumbar spine and a hip negatively correlated with OC and β-CTX. The same correlation between OC and bone density was observed [45] when bone density was expressed as the *Z* value.

When serum sclerostin concentrations were correlated with thyroid hormones and the BMD measurement, a positive association was found only with FT3 at hyperthyroid state. These findings differ from some previous data where a negative correlation between sclerostin and BMD was found in hyperthyroid mice in which the fourth lumbar vertebra, distal femur, and femoral shaft were analyzed with μCT [23]. In one study involving postmenopausal women, a negative association between sclerostin and BMD of the femoral neck, trochanter, and total hip was noted [46]. On the other hand, a positive association between sclerostin levels and BMD of the lumbar spine was found in a study including patients on peritoneal dialysis [47] and postmenopausal women [48].

At the euthyroid state, a positive association between FT3 and DKK1, as well as a negative correlation between TSH and DKK1, was present. However, there was no association between DKK1 and BMD measurements as well as other bone turnover markers. This is the first time that correlation analysis of DKK1 with thyroid hormones was investigated, reporting the existence of cross-talk between Wnt and the thyroid hormone pathway in bone tissue. Still, postnatal deletion of DKK1 in mice did not reverse bone tissue changes induced by hyperthyroidism [24]. Therefore, it is more likely that serum concentration changes of these two Wnt inhibitors are primarily induced by the pathophysiological impact of thyroid hormones on the Wnt pathway and themselves, thus influencing bone remodeling process.

The main limitation of our study is the relatively small sample size. In addition, patients of both genders, as well as pre- and post-menopausal women, were included in the study. It would be interesting to investigate whether there are differences in Wnt inhibitor concentrations depending on gender and depending on the age and menopause status in these patients. Further research is needed, including a larger number of patients to provide additional insights into the role of Wnt inhibitors in the bone metabolism of hyperthyroid patients. Still, this is a first prospective study investigating the role of sclerostin and DKK1 in patients newly diagnosed with GD and treated with ATD.

## 4. Materials and Methods

### 4.1. Study Design and Patients

This was a prospective longitudinal cohort study that enrolled 37 patients treated at the Clinical Institute of Nuclear Medicine and Radiation Protection, University Hospital Osijek.

The study protocol was approved by the Ethical Committee of the Clinical Hospital Center Osijek (Ethical Approval Code: R2-6782/2018.) and by the Ethical Committee for Research at University J.J. Strossmayer, Faculty of Medicine Osijek (Ethical Approval Code: 2158-61-07-17-214). All patients gave written informed consent before being included in the study.

Participants were newly diagnosed patients with GD enrolled in the period from December 2017 to January 2020. They were treated with ATD and followed-up for one year.

Exclusion criteria were: history of prior osteoporosis treatment, prior treatment with medications that interfere with bone metabolism such as chronic administration of corticosteroids (>3 months duration) and hormone replacement therapy (HRT), primary hyperparathyroidism, Cushing’s disease, cardiac disease, inflammatory bowel disease (IBD), malabsorption, acute infection, chronic kidney, gastrointestinal or liver disease, ankylosing spondylitis, carcinoma, toxic nodular goiter, toxic thyroid adenoma, long term immobilization, GD relapse, drug-induced thyrotoxicosis, subacute, silent or postpartum thyroiditis, women who gave birth and/or nursed a year before GD diagnosis, subclinical hyperthyroidism, and hashitoxicosis and thyroid ophthalmopathy requiring corticosteroid therapy or thyroid operation according to EUGOGO guidelines [49].

### 4.2. Study Protocol

Data and measurements were obtained at baseline and a control visit after one year. At the baseline visit, patients’ anthropometrical parameters were obtained, and BMI was calculated. Blood and urine samples were taken at both time points to determine FT4, FT3, TSH and anti-TSH-R, OC, β-CTX, DPD, vitamin D, sclerostin, and DKK1. DXA measurements of the lumbar spine, total hip, and femoral neck were performed at both visits.

Blood samples were drawn between 8 a.m. and 10 a.m. after overnight fasting. Blood samples for sclerostin and DKK1 were centrifuged and aliquots of serum were stored at −80 °C until analyzed.

FT4, FT3, and TSH concentrations were measured by chemiluminescent immunoassay using commercial kits (Abbott, IL, USA). The normal reference range of FT4 was 9.01–19.05 pmol/L; for FT3, 2.63–5.70 pmol/L; and TSH, 0.350–4.940 mIU/L. Anti-TSH-R was measured by an electrochemiluminescent immunoassay using a commercial kit (Roche, Mannheim, Germany), and the normal reference range was positive >1.75 U/L. OC and β-CTX were measured by a chemiluminescent immunoassay using commercial kits (Roche, Mannheim, Germany). The normal reference range of OC was: pre-menopause 11–43 μg/L, post-menopause 15–46 μg/L, osteoporosis 13–48 μg/L. The normal reference range of β-CTX was: pre-menopause, 0.025–0.575 μg/L and post-menopause, 0.100–1.000 μg/L. DPD was measured by chromatography using commercial kit (Chromsystems Instruments, Gräfelfing Germany). The normal reference range was 3.0–7.4 nmol. Vitamin D was measured by chromatography using a commercial kit (RECIPE Chemicals, Munich, Germany). The normal reference range was 20.0–100.0 ug/L.

Serum concentrations of sclerostin were measured using a human sclerostin enzyme-linked immunosorbent assay (ELISA) kit (Human SOST ELISA Kit, Sigma, Saint Louis, MO, USA), and serum DKK1 concentrations were measured using a DKK1 ELISA kit (Human DKK1 ELISA Kit, Cusabio, Wuhan, China) according to the manufacturer’s instructions.

BMD was measured by DXA imaging at the lumbar spine, total hip, and femoral neck on a Lunar Prodigy Primo device (GE Healthcare, New York, NY, USA) according to the manufacturer’s operating protocol.

### 4.3. GD Diagnosis and Treatment

GD diagnosis was based on the elevated serum concentrations of FT4 and FT3, suppressed serum concentration of TSH, elevated concentrations of anti-TSH-R, diffuse thyroid accumulation of Tc-99m pertechnetate on scintigraphy, and ultrasound characteristics of GD.

The first line of treatment was thiamazole with a 30–60 mg daily dose divided into three doses. Propylthiouracil (PTU) was introduced as a second line of treatment in the case of allergy or side effects on therapy with thiamazole. The first checkup was scheduled after four weeks and the dose of the medication was adjusted if needed, whereas any subsequent checkup and dose adjustment was scheduled individually for each patient. The maintenance dosage was 2.5–10 mg thiamazole daily or 50–100 mg PTU daily.

### 4.4. Statistical Analysis

Categorical data are represented by absolute and relative frequencies. Numerical data are described as median and interquartile ranges. Categorical variable differences at the baseline and control points were calculated by the McNemar–Bowkers test and marginal homogeneity test. The distribution normality of numerical variables was tested by Shapiro–Wilks test. Numerical variable differences between the two measurements were tested by the Wilcoxon test in the case of normal distribution. If the distribution was not normal, the correlation of numerical variables was assessed by Spearman’s correlation coefficient ρ (rho), as well as partial correlation (adjusted for gender, age, and vitamin D intake). Logistic regression analysis was used to determine the influence of individual predictors for osteopenia/osteoporosis onset (adjusted for age, gender, BMI, and vitamin D intake). All *p* values were two-sided. The significance level was set to α = 0.05. MedCalc^®^ Statistical Software version 20.023 (MedCalc Statistical Software version 20.111, MedCalc Software Ltd., Ostend, Belgium) and IBM SPSS Statistics for Windows (version 23.0., NY: IBM Corp., Armonk, NY, USA) were used for statistical analysis. For the observation of major differences with the significance level of 0.05, medium effect 0.05, and 80% statistical power, a minimum of 34 participants was needed according to G*Power version 3.1.2 analysis program.

## 5. Conclusions

In this study we have demonstrated opposite changes in serum concentrations of sclerostin and DKK1 along with changes in thyroid hormones from hyperthyroid to the euthyroid state. Sclerostin levels decreased while DKK1 concentrations increased after ATD treatment and euthyroid state achievement, accompanied by improvements in the lumbar spine, hip bone density, biomarkers of bone remodeling OC, β-CTX, and DPD. Furthermore, positive association between sclerostin and FT3 in a hyperthyroid state was observed, as well as a positive correlation between DKK1 and FT3 and negative correlation between DKK1 and TSH in a euthyroid state. Thus, we could assume that sclerostin and DKK1 levels are affected by thyroid status, indicating involvement in pathophysiological processes activated in GD via the canonical Wnt pathway; however, those changes are reversible with the timely initiation of ATD therapy. Hence, a timely diagnosis of GD is essential to start treatment and prevent the accumulated effects of other risk factors influencing bone health, such as age and sex.

## Figures and Tables

**Table 1:** Baseline characteristics of subjects.

	Median (Interquartile Range)	Minimum–Maximum
Age (year)	47 (33–58)	22–74
Height (cm)	165 (160–170)	147–184
Weight (kg) BMI (kg/m^2^)	67 (54.5–71) 24.4 (20.25–26.95)	50–92 17.9–33.9
Vitamin D ug/L	18 (10.9–18.3)	

BMI-body mass index.

**Table 2:** Changes in concentration levels of thyroid hormones, TSH, and anti-TSH-R after one year of ATD treatment.

	Median (Interquartile Range)		Difference (95% Reliability Range)	*p* *
	Baseline Point	Control Point		
FT4 pmol/L	31.85 (29.27–39.41)	11.45 (10.73–12.76)	−21.9 (−24.2 to −19.2)	<0.001
FT3 pmol/L	16.3 (12.72–26.85)	3.98 (3.61–4.47)	−15.3 (−19.7 to −11.2)	<0.001
TSH mIU/L	0.01 (0.01–0.01)	1.76 (1.06–3.02)	1.99 (1.47 to 2.68)	<0.001
Anti-TSH-R U/L	8.7 (4.15–13.45)	1.1 (0.45–2.4)	−8.25 (−10.25 to −5.2)	<0.001

* Wilcoxon test; ATD—antithyroid drugs FT4—free thyroxine; FT3—free triiodothyronine; TSH—thyroid-stimulating hormone; Anti-TSH-R—anti-thyroid stimulating hormone receptor antibody.

**Table 3:** Changes in bone remodeling markers and Wnt inhibitors after one year of ATD treatment.

	Median (Interquartile Range)		Difference (95% Reliability Range)	*p* *
	Baseline Point	Control Point		
β-CTX μg/L	1.21 (0.79–1.7)	0.37 (0.24–0.51)	−0.89 (−1.11 to −0.67)	<0.001
OC μg/L	58.5 (47.5–87.5)	26 (20.5–35.5)	−35.3 (−45 to −25.5)	<0.001
DPD nmol	19.35 (12.3–25.5)	4.05 (3–4.95)	−14.9 (−18.5 to −11.6)	<0.001
Sclerostin pg/mL	134.68 (30.3–306.9)	65.82 (27.7–177.7)	−34.3 (−86.7 to −6.5)	<0.001
DKK1 pg/mL	0.403 (0.265–0.595)	0.604 (0.410–0.889)	0.181 (0.036 to 0.321)	0.01

* Wilcoxon test; ATD—antithyroid drugs; β-CTX–Beta-Cross Laps; OC–osteocalcin; DPD–deoxypyridinoline; DKK1–Dickkopf-1.

**Table 4:** Changes in DXA measurements of the lumbar spine, total hip, and femoral neck after one year of ATD treatment.

Participants DXA Results	Median (Interquartile Range)		Difference (95% Reliability Range)	*p* *
	Baseline Point	Control Point		
T-score L1–L4	−1.3 (−1.9 to 0.3)	−0.7 (−1.4 to 0.7)	0.4 (0.2 to 0.65)	0.001
T-score of the hip	−0.6 (−1.3 to −0.10)	−0.3 (−1.025 to 1.25)	0.5 (0.3 to 0.7)	<0.001
T-score of the femoral neck	−0.7 (−1.63 to 0.23)	−0.4 (−1.03 to 0.425)	0.35 (0.2 to 0.5)	<0.001
L1–L4 BMD g/cm^2^	1.016 (0.946 to 1.21)	1.095 (1.01 to 1.27)	0.067 (0.04 to 0.105)	<0.001
Hip BMD g/cm^2^	0.915 (0.84 to 0.963)	0.963 (0.879 to 1.148)	0.079 (0.049 to 0.115)	<0.001
Femoral neck BMD g/cm^2^	0.894 (0.78 to 0.946)	0.935 (0.852 to 1.032)	0.062 (0.036 to 0.089)	<0.001

* Wilcoxon test; ATD—antithyroid drugs; BMD—bone mineral density; DXA—dual-energy X-ray absorptiometry.

**Table 5:** Changes in the lumbar spine and hip bone density after one year of ATD treatment.

		Participants Number (%)				*p*
		DXA Results According to T-Score at Baseline Point				
		Normal	Osteopenia	Osteoporosis	Total	
Lumbar spine						
Participants number (%) Control Point	Normal bone density	15	10	0	25 (68)	0.007 *
	Osteopenia	1	10	0	11 (30)	
	Osteoporosis	0	0	1	1 (3)	
	Total	16 (43)	20 (54)	1 (3)	37 (100)	
Hip						
Participants number (%) Control point	Normal	23	4	0	27 (73)	0.10 **
	Osteopenia	1	8	1	10 (27)	
	Osteoporosis	0	0	0	0	
	Total	24 (65)	12 (32)	1 (3)	37 (100)	

* McNemar–Bowkers test; ** Test of marginal homogeneity. ATD—antithyroid drugs; DXA—dual-energy X-ray absorptiometry.

**Table 6:** Association of thyroid hormones, TSH, anti-TSH-R, and DXA findings with markers of bone turnover and Wnt inhibitors at baseline.

Baseline Point	Spearman’s Correlations Coefficient Rho (*p*-Value)				
	OC μg/L	DPD nmol	β-CTX μg/L	Sclerostin pg/mL	DKK 1 pg/mL
FT4 pmol/L	0.377 (0.02)	0.581 (<0.001)	0.360 (0.03)	0.271 (0.10)	0.208 (0.22)
FT3 pmol/L	0.355 (0.04)	0.694 (<0.001)	0.394 (0.02)	0.226 (0.19)	0.259 (0.13)
TSH mIU/L					
Anti-TSH-R U/L	0.166 (0.33)	0.404 (0.01)	−0.002 (0.99)	0.138 (0.42)	0.284 (0.09)
L1–L4 T-score	−0.447 (0.006)	−0.058 (0.73)	−0.396 (0.02)	−0.158 (0.35)	−0.235 (0.16)
Hip T-score	−0.496 (0.002)	−0.106 (0.53)	−0.393 (0.02)	−0.090 (0.60)	−0.270 (0.11)
Femoral neck T-score	−0.406 (0.01)	−0.119 (0.48)	−0.374 (0.03)	−0.262 (0.12)	−0.244 (0.15)
L1–L4 BMD g/cm^2^	−0.407 (0.01)	−0.034 (0.84)	−0.385 (0.02)	−0.114 (0.50)	−0.300 (0.07)
Hip BMD g/cm^2^	−0.439 (0.007)	−0.103 (0.54)	−0.391 (0.02)	−0.072 (0.67)	−0.229 (0.17)
Femoral neck BMD g/cm^2^	−0.379 (0.02)	−0.153 (0.37)	−0.348 (0.04)	−0.193 (0.25)	−0.308 (0.06)

DXA-dual-energy X-ray absorptiometry; FT4-free thyroxine; FT3-free triiodothyronine; TSH-thyroid-stimulating hormone; Anti-TSH-R—anti-thyroid stimulating hormone receptor antibodies; BMD-bone mineral density.

**Table 7:** Association of thyroid hormones, TSH, anti-TSH-R, and DXA findings with markers of bone turnover and Wnt inhibitors on control measurement at control point.

Control Point	Spearman’s Correlations Coefficient Rho (*p*-Value)				
	OC μg/L	DPD nmol	β-CTX μg/L	Sclerostin pg/mL	DKK 1 pg/mL
FT4 pmol/L	0.023 (0.89)	0.478 (0.003)	0.042 (0.81)	−0.246 (0.14)	0.319 (0.05)
FT3 pmol/L	−0.064 (0.71)	0.178 (0.30)	−0.017 (0.92)	−0.034 (0.84)	0.480 (0.003)
TSH mIU/L	0.121 (0.48)	−0.325 (0.05)	0.117 (0.49)	0.136 (0.42)	−0.335 (0.04)
Anti-TSH-R U/L	−0.122 (0.47)	−0.248 (0.14)	−0.111 (0.51)	0.261 (0.12)	−0.219 (0.19)
L1–L4 T-score	−0.354 (0.03)	−0.134 (0.43)	−0.445 (0.006)	0.142 (0.40)	−0.257 (0.12)
Hip T-score	−0.449 (0.002)	0.135 (0.43)	−0.239 (0.15)	−0.091 (0.59)	−0.157 (0.35)
Femoral neck T-score	−0.484 (0.002)	0.095 (0.58)	−0.318 (0.05)	−0.143 (0.40)	−0.111 (0.51)
L1–L4 BMD g/cm^2^	−0.290 (0.08)	−0.117 (0.5)	−0.391 (0.02)	0.025 (0.88)	−0.223 (0.18)
Hip BMD g/cm^2^	−0.461 (0.004)	0.073 (0.67)	−0.269 (0.11)	−0.099 (0.56)	−0.197 (0.24)
Femoral neck BMD g/cm^2^	−0.524 (0.001)	0.02 (0.91)	−0.354 (0.03)	−0.185 (0.27)	−0.179 (0.29)

DXA-dual-energy X-ray absorptiometry; FT4—free thyroxine; FT3—free triiodothyronine; TSH—thyroid-stimulating hormone; Anti-TSH-R—anti-thyroid stimulating hormone receptor antibodies; BMD—bone mineral density.

## Data Availability

Data available on request due to restrictions eg privacy or ethical. The data presented in this study are available on request from the corresponding author. The data are not publicly available due to privacy.
